# Meiosis-Based Laboratory Evolution of the Thermal Tolerance in *Kluyveromyces marxianus*

**DOI:** 10.3389/fbioe.2021.799756

**Published:** 2022-01-11

**Authors:** Li Wu, Yilin Lyu, Pingping Wu, Tongyu Luo, Junyuan Zeng, Tianfang Shi, Jungang Zhou, Yao Yu, Hong Lu

**Affiliations:** ^1^ State Key Laboratory of Genetic Engineering, School of Life Sciences, Fudan University, Shanghai, China; ^2^ Shanghai Engineering Research Center of Industrial Microorganisms, Shanghai, China; ^3^ Shanghai Collaborative Innovation Center for Biomanufacturing Technology, Shanghai, China

**Keywords:** Kluyveromyces marxianus, thermal tolerance, laboratory evolution, meiosis, iterative cycle, PSR1, PDE2

## Abstract

*Kluyveromyces marxianus* is the fastest-growing eukaryote and a promising host for producing bioethanol and heterologous proteins. To perform a laboratory evolution of thermal tolerance in *K. marxianus*, diploid, triploid and tetraploid strains were constructed, respectively*.* Considering the genetic diversity caused by genetic recombination in meiosis, we established an iterative cycle of “diploid/polyploid - meiosis - selection of spores at high temperature” to screen thermotolerant strains. Results showed that the evolution of thermal tolerance in diploid strain was more efficient than that in triploid and tetraploid strains. The thermal tolerance of the progenies of diploid and triploid strains after a two-round screen was significantly improved than that after a one-round screen, while the thermal tolerance of the progenies after the one-round screen was better than that of the initial strain. After a two-round screen, the maximum tolerable temperature of Dip2-8, a progeny of diploid strain, was 3°C higher than that of the original strain. Whole-genome sequencing revealed nonsense mutations of *PSR1* and *PDE2* in the thermotolerant progenies. Deletion of either *PSR1* or *PDE2* in the original strain improved thermotolerance and two deletions displayed additive effects, suggesting *PSR1* and *PDE2* negatively regulated the thermotolerance of *K. marxianus* in parallel pathways. Therefore, the iterative cycle of “meiosis - spore screening” developed in this study provides an efficient way to perform the laboratory evolution of heat resistance in yeast.

## Introduction

The mutation is the major driving force of the adaptive evolution, leading to traits in coping with various environmental stress, including heat, salinity, alkalinity, acidity and so on (Wright, 2004). Heat resistance, also called thermal tolerance, is well-recognized as a quantitative trait controlled by a range of genes and pathways (Gao et al., 2016). For example, mutations of Rho1-Pkc1 pathway increase the thermal tolerance by affecting the integrity of the cell wall (Huang et al., 2018). Glutathione directly reduces the hydroxyl radical to form H_2_O, thereby removing oxygen free radicals in cells, to increase the heat resistance of cells (Grant et al., 1996). Trehalose mainly protects cells from high-temperature damage by stabilizing the cell membrane structure and maintaining the conformation of intracellular proteins (Martinez-Esparza et al., 2011).

*Kluyveromyces marxianus* is a food-grade yeast commonly isolated in dairy environments. *K. marxianus* is the fastest-growing eukaryote reported so far, with a specific growth rate of 0.7–0.99 h^−1^ (Groeneveld et al., 2009). *K. marxianus* displays superior thermotolerance, as it can grow at 45°C and tolerate temperatures over 50°C, making it a promising platform for the production of bioethanol and chemicals (Lane and Morrissey, 2010; Gombert et al., 2016). Same as *Saccharomyces cerevisiae*, *K. marxianus* is a homothallic yeast with two mating types, *MAT*a and *MAT*α. Haploid cells switch the mating type spontaneously and haploid cells are capable of mating with other haploid cells of the opposite mating type to produce *MAT*a/α diploids (Cernak et al., 2018). When encountering adversities unsuitable for growth, diploid cells tend to undergo meiosis to produce tetrad. During meiosis, chromosomal crossover occurs, generating novel allele combinations, and some of which may help cells through harsh environments and subsequently promote the evolution of stress resistance (Owens et al., 2018). By combining meiosis and screen, the researcher successfully increased the ethanol production of *S. cerevisiae* by 10.96% (Hou, 2010). In our previous study, a 5-fold increase in the production of ferulic acid lipase was achieved by performing meiosis of *K. marxianus* diploids and subsequent screening of high-yield spores (Wu et al., 2020a). To our knowledge, there is no report of meiosis-mediated evolution of thermal tolerance in yeast yet.

Besides diploid, triploid and tetraploid can also undergo meiosis. In the meiosis of the triploids, homologous chromosomes cannot be distributed equally, resulting in aneuploidy (Charles et al., 2010). In the meiosis of tetraploids, there was an increased chance of asymmetric separation of homologous chromosomes, resulting in progeny cells of multiple ploidies (Loidl, 1995). Therefore, the genetic diversity of spores generated by triploids and tetraploids was expected to be different from that of spores generated by diploids, which might provide a valuable source for the screen of desired phenotypes. However, the triploid and tetraploid strain of *K. marxianus* has not been built in the laboratory yet.

In this study, triploid and tetraploid cells were constructed from diploid *K. marxianus* cells. Cells were screened by an iterative cycle of “diploid/polyploid - meiosis - selection of spores at high temperature”. After a two-round screen, progenies displaying significantly enhanced thermal tolerance were obtained. Whole-genome sequencing suggested *PSR1* and *PDE2* were negative regulators of the thermotolerance. This study provides the first meiosis-based iterative cycle for the evolution of thermal tolerance in yeast. The method can be applied in the laboratory evolution of resistance to other stresses.

## Materials and Methods

### Strains and Plasmids

Strains used in this study were listed in Table 1. Wild-type *K. marxianus* strain FIM1 was deposited in China General Microbiological Culture Collection Center (CGMCC No.10621). *URA3* was deleted in FIM1 to obtain FIM1Δ*u* as described before (Zhou et al., 2018). In FIM1Δ*u*, the *HML* locus and *TRP1* locus were deleted to obtain a-FIM1Δ*u*Δ*w*, the *HMR* locus and *HIS3* were deleted to obtain α-FIM1Δ*u*Δ*h*, *PSR1* and *PED2* were deleted to obtain FIM1-*psr1*Δ and FIM1-*pde2*Δ, respectively. *PED2* was deleted in FIM1-*psr1*Δ to obtain FIM1-*psr1*Δ*pde2*Δ. Genes were deleted by CRISPR/Cas9 as described before (Liu et al., 2018). Primers and plasmids used in the construction were shown in Supplementary Tables S1 and S2.

**TABLE 1:** Strains used in this study.

Names	Genotypes	Sources
FIM1		Zhou et al. (2018)
FIM1Δ*u*	*ura3*Δ	Zhou et al. (2018)
a-FIM1Δ*u*Δ*w*	*MAT*a *ura3*Δ *hml*Δ *trp1*Δ	This study
α-FIM1Δ*u*Δ*h*	*MAT*α *ura3*Δ *hmr*Δ *his3*Δ	This study
KM-Diploid-S	*MAT*a/α *ura3*Δ	This study
KM-Diploid	*MAT*a/α *ura3*Δ *hml*Δ *hmr*Δ	This study
KM-Diploid-*MAT*a/a	*MAT*a/a *ura3*Δ *hml*Δ *hmr*Δ	This study
KM-Diploid-*MAT*α/α	*MAT*α/α *ura3*Δ *hml*Δ *hmr*Δ	This study
KM-Triploid	*MAT*a/a/α *ura3*Δ *hml*Δ *hmr*Δ	This study
KM-Tetraploid	*MAT*a/a/α/α *ura3*Δ *hml*Δ *hmr*Δ	This study
FIM1-*psr1*Δ	*ura3*Δ *psr1*Δ	This study
FIM1-*pde2*Δ	*ura3*Δ *pde2*Δ	This study
FIM1-*psr1*Δ*pde2*Δ	*ura3*Δ *psr1*Δ *ped2*Δ	This study

a-FIM1Δ*u*Δ*w* mated with α-FIM1Δ*u*Δ*h* to form KM-Diploid-S (*MAT*a/α, *ura3*Δ) as described before (Wu et al., 2020b). The *HML* and *HMR* locus of KM-Diploid-S were deleted by CRISPR/Cas9 to obtain KM-Diploid. The primer pairs W29F/W29R and W30F/W30R were used to identify the deletion of *HMR* or *HML* locus. The mating type of KM-Diploid (*MAT*a/α) was switched to *MAT*a/a by replacing *MAT*α locus with *MAT*a using CRISPR/Cas9, and the resultant strain was KM-Diploid-*MAT*a/a. Similarly, the mating type of KM-Diploid was switched to *MAT*α/α by replacing *MAT*a locus with *MAT*α, and the resultant strain was KM-Diploid-*MAT*α/α.

To construct the tetraploid strain, a pKD1-based plasmid carrying *KanMX6* (pUKDN127-Kan) was transformed into KM-Diploid-*MAT*a/a strain, and a pKD1-based plasmid carrying *hphMX4* (pUKDN127-Hyg) was transformed into KM-Diploid-*MAT*α/α strain. Transformants were cultured separately in YPD plates (10 g/L yeast extract, 20 g/L hipolypepton, 20 g/L glucose, 20 g/L agar) overnight. Cells were mixed and spread onto ME medium (50 g/L maltose extract, 30 g/L agar) to mate. Cells were then cultured at 30°C for 2 days and selected on YPD + G418 (0.2 mg/ml) +Hygromycin (0.25 mg/ml) plates to obtain tetraploid strain KM-tetraploid. The triploid strain was constructed similarly. KM-Diploid-*MAT*a/a cells transformed with pUKDN127-Kan were mated with α-FIM1Δ*u*Δ*h* cells transformed with pUKDN127-Hyg. Triploid strain KM-triploid was selected on the YPD + G418 + Hygromycin plate.

### Meiosis and Selection of Spores at High Temperature

Diploid, triploid or tetraploid cells were cultured in 3 ml YPD liquid medium (10 g/L yeast extract, 20 g/L hipolypepton, 20 g/L glucose) at 30°C for 12 h. Then cultures were inoculated into 50 ml YPA liquid medium (10 g/L potassium acetate, 20 g/L hipolypepton, 10 g/L yeast extract) at an initial optical density at 600 nm (OD_600_) of 0.1, and cultured at 30°C for 6–8 h till OD_600_ reached 1.0–1.2. Cells were centrifuged and washed twice with sterile water. Cells were resuspended in 50 ml 2% potassium acetate (KAc) and cultured at 30°C for 24 h to initiate meiosis and sporulation. 1 ml sample was centrifuged and washed twice with sterile water. The cells were resuspended in 500 μl sterile water and then treated with 25 μl zymolyase (5 U/μl, E1004, Zymoresearch, United States) and 5 μl β-mercaptoethanol at 4°C for 24 h. The sample was supplemented with 200 μl 1.5% NP-40 and incubated at 30°C for 30 min to lyse vegetative cells. Cells were sonicated for 30 s times (Bioruptor UCD-300, Diagenode, Belgium), and then spread to YPD plates. For the first round of screen, spores generated by KM-Diploid, KM-Triploid and KM-tetraploid cells were grown at 43°C for 2 days. A total of 392 clones formed on the plates were selected and grown at 43°C in 3 ml YPD liquid medium for 4 h. Then, 3 μl culture was spotted onto YPD plates and grown at 43, 43, 45, and 46°C for 2 days. For the second-round screen, spores generated by diploid 2–2 and triploid 3–2 were grown at 46°C for 2 days. A total of 580 clones from 2–2 and 680 clones from 3–2 formed on the plates were selected and grown at 46°C in 3 ml liquid YPD for 4 h. Then, 3 μl culture was spotted onto YPD plates and grown at 45, 46, 47, and 48 for 2 days. Substantial growth on the plates indicated thermal tolerance to the temperature.

### Determination of Mating Type and Auxotrophic Markers

The mating types were determined by PCR using three primers (YY270F, YY271F, YY272F). *MAT*a locus produced a band of 1,062 bp and *MAT*α locus produced a band of 1,515 bp. Cells failing to grow on the SC-his (20 g/L glucose, 6.7 g/L yeast nitrogen base, 40 mg/L uracil, 40 mg/L leucine, 40 mg/L tryptophan, 20 g/L agar) and SC-trp plate (20 g/L glucose, 6.7 g/L yeast nitrogen base, 40 mg/L histidine, 40 mg/L leucine, 40 mg/L uracil, 20 g/L agar) carried *his3*Δ and *trp1*Δ auxotrophic markers, respectively. Primers were listed in Supplementary Table S1.

### Flow Cytometry

Cells were grown in YPD overnight. Cells of 800 μl cultures were pelleted and washed twice by 1 ml phosphate buffer (0.2 M Na_2_HPO_4_, pH adjusted to 7.0 by 0.1 M citric acid). Cells were resuspended gently in 1 ml of cold 75% ethanol and stored at 4°C for 5 h. Cells were washed by the phosphate buffer once and then resuspended in 800 μl phosphate buffer. The sample was supplemented with 10 μL RNase (50 mg/ml) and incubated at 37°C for 24 h. Cells were pelleted and resuspended in 1 ml phosphate buffer. Cells were supplemented with 5 μl propidium iodide solution (10 μg/ml propidium iodide in phosphate buffer) and stained for 30 min in dark. Cells were sonicated for 30 s 3 times. 10,000 cells were measured by a FACS Calibur flow cytometer (Becton Dickinson, United States) and data were analyzed by Flowjo 2.0.

### Spot Assay

For spot assays of FIM1, KM-Diploid, KM-Triploid, KM-tetraploid, FIM1-*psr1*Δ, FIM1-*pde2*Δ and FIM1-*psr1*Δ*pder2*Δ, cells were grown in 3 ml YPD liquid medium at 30°C for 12 h. For the spot assay of spores, individual spore was grown in 3 ml YPD liquid medium and grown at 43°C or 46°C for 12 h. The culture was adjusted to an OD_600_ of 0.6 and then diluted fivefold five times. 3 μl dilutions were spotted on YPD plates. Plates were incubated at 30–48°C.

### Growth Curves

Cells were grown in 3 ml YPD liquid medium at 30°C for 12 h and then diluted into 50 ml fresh YPD liquid medium to start at an OD_600_ of 0.01. Cells were grown at 47°C for 120 h. The OD_600_ of the culture was measured every 6 or 12 h. The experiment was performed with three parallel cultures.

### Whole-Genome Sequencing

Cells were grown in 3 ml YPD liquid medium at 30°C for 12 h. Genomic DNA was extracted by a Yeast Genomic DNA Extraction kit (D1900, Solarbio, China). Whole-genome sequencing was performed by Illumina Hiseq (Mingma technologies, Shanghai, China). Significant SNPs and InDELs were identified by sequence alignment using the genome of FIM1 as a reference (Yu et al., 2021).

## Results

### Improving the Thermal Tolerance of *Kluyveromyces marxianus* by Meiosis-Based Iterative Screen

**FIGURE 1:**
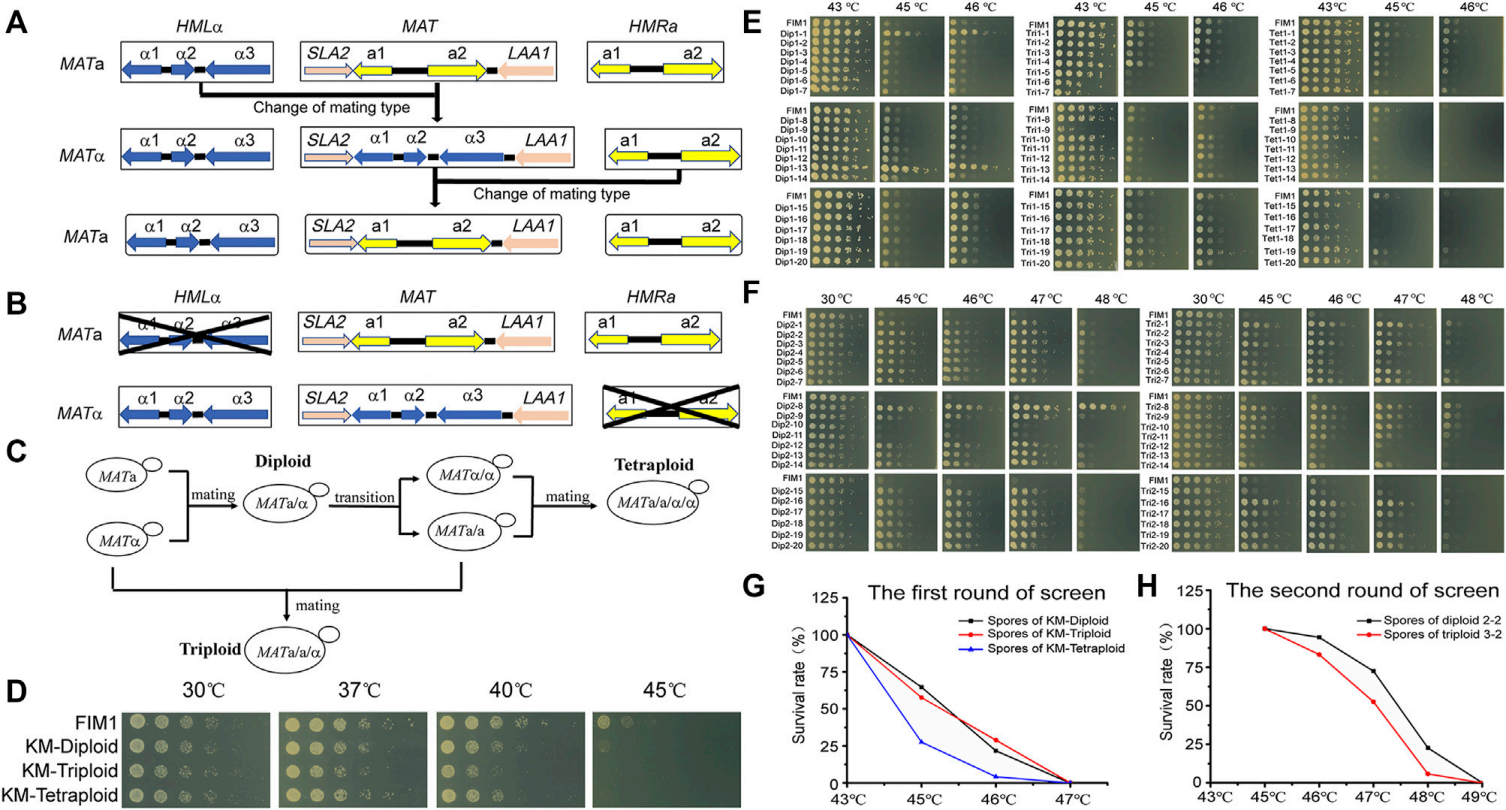
The meiosis-based evolution of the thermotolerance in *K. marxianus*. **(A)** Switches of mating type in *K. marxianus*. **(B)** Constructions of stable *MAT*a and *MAT*α cells. **(C)** Constructions of diploid, triploid and tetraploid cells. **(D)** Spot assay of FIM1, KM-Diploid, KM-Triploid and KM-Tetraploid strains at different temperatures. **(E)** Spot assay of spores generated by KM-Diploid, KM-Triploid and KM-Tetraploid at different temperatures. **(F)** Spot assay of spores generated by 2–2 and 3–2 at different temperatures. **(G, H)** Survival rates of spores generated in the first **(G)** and second **(H)** rounds of the screen at different temperatures.

The mating type of *K. marxianus* cells is determined by genes located in the *MAT* locus. The mating-type switches in *K. marxianus* are expected to occur spontaneously in the same way as reported in *Kluyveromyces lactis*, during which sequence of *MAT* locus was replaced by that of *HML* locus carrying silent *MAT*α information or by that of *HMR* locus carrying silent *MAT*a information, in a manner independent of homothallic switching (HO) endonuclease (Barsoum et al., 2010; Lee et al., 2018) (Figure 1A). To prevent the switch of mating type in this study, *HML* locus was deleted in *MAT*a cells and *HMR* locus was deleted in *MAT*α cells to obtain stable *MAT*a (a-FIM1Δ*u*Δ*w*) and *MAT*α (α-FIM1Δ*u*Δ*h*) haploid cells, respectively (Figure 1B).

a-FIM1Δ*u*Δ*w* mated with α-FIM1Δ*u*Δ*h* to form diploid (KM-Diploid-S), in which *HML* and *HMR* loci were deleted subsequently to form stable diploid strain (KM-Diploid). The mating type of KM-Diploid (*MAT*a/α) was switched to *MAT*a/a and *MAT*α/α, respectively. *MAT*a/a cells mated with *MAT*α and *MAT*α/α cells, to obtain triploid *MAT*a/a/α and tetraploid *MAT*a/a/α/α cells, respectively (Figure 1C). Details of the strain construction were described in methods. Ploidies were determined by flow cytometry. DNA contents of haploid, diploid, triploid and tetraploid strains were consistent with theoretical values (Supplementary Figure S1A).

The effect of the ploidy on the thermal tolerance was investigated by a spot assay in the first place. The result showed that ploidy has little effect on cell growth under 30 and 37°C. The growth of diploid, triploid and tetraploid cells was poorer than that of haploid cells at 40°C. Haploid cells displayed limited growth at 45°C, while diploid, triploid and tetraploid cells could not grow at this temperature (Figure 1D). The result suggested, in *K. marxianus*, cells of higher ploidy levels, including diploid, triploid and tetraploid cells, displayed reduced thermotolerance than haploid cells. A similar result was reported in *S. cerevisiae*, as the thermotolerance of diploid and tetraploid cells at 50°C declined compared to that of haploid cells after a pre-heat shock (Piper et al., 1987).

Diploid, triploid and tetraploid cells underwent meiosis and produced spores in 2% KAc (Supplementary Figure S1B). Spores were grown at 43°C for 2 days till thermotolerant spores formed clones on the plates. A total of 392 clones generated by diploid, triploid or tetraploid were selected and grown at 43, 45, and 46°C for 2 days. Less than half of the clones sporulated from diploid, triploid and tetraploid strains could grow at 46°C (Supplementary Figure S2). Twenty spores displaying the best thermotolerance in the progenies of diploid, triploid and tetraploid strains were named Dip1-1∼20, Tri1-1∼20 and Tet1-1∼20, respectively. The thermotolerance of these spores was investigated by a spot assay (Figure 1E). Among the spores generated by KM-Diploid, Dip1-1 and Dip1-13 displayed better growth than wild-type FIM1 strain, while the thermal tolerance of the rest 18 spores was similar to that of the FIM1 strain. In the spores generated by KM-Triploid, Tri1-19 displayed better thermal tolerance than FIM1. Five spores, including Tri1-5, 6, 7, 9, 15 displayed worse thermal tolerance than FIM1, while the rest spores were similar to FIM1. In the spores generated by KM-Tetraploid, only Tet1-1 and Tet1-19 displayed similar thermal tolerance as FIM1, while the thermal tolerance of the rest clones was worse than that of FIM1. Results suggested that in the first round of “meiosis - selection of spores at high temperature”, the frequency of producing thermotolerant spores from the diploid strain was higher than that from triploid and tetraploid strains.

To find compatible pairs to construct diploid and polyploid strains in the second round of screen, the auxotrophic markers (Δ*trp*1 and Δ*his*3), the mating types and ploidies of spores were investigated (Supplementary Figure S3). To evaluate the effect of ploidy on the efficiency of evolution, spores generated by the diploid in the first round of screen were selected to construct the initial diploid strain for the second round of screen. Similarly, spores generated by the triploid were selected to construct triploid, and those by tetraploid were used to construct tetraploid. Based on the above considerations, Dip1-1(*MAT*α, *ura3*Δ) was selected to mate with Dip1-13 (*MAT*a, *ura3*Δ*his3*Δ) to form diploid 2-2 (*MAT*a/α, *ura3*Δ). Tri1-19 (*MAT*α, *ura3*Δ) mated with Tri1-5 (*MAT*a/a, *ura3*Δ) to form triploid 3-2 (*MAT*a/a/α, *ura3*Δ). Tet1-1 (*MAT*a/a, *ura3*Δ) mated with Tet1-15 (*MAT*α/α, *ura3*Δ*his3*Δ) to form tetraploid 4-2 (*MAT*a/a/α/α, *ura3*Δ). The flow cytometry analysis results showed that the DNA contents of 2–2, 3–2 and 4–2 were consistent with their expected ploidies (Supplementary Figure S4A).

In the second round of screen, 2–2, 3–2 and 4–2 were cultured in 2% KAc to produce spores (Supplementary Figure S4B). First, a portion of spores was grown at 43, 45, 46, and 47°C for 2 days. Spores of 2–2 and 3–2 could grow at 46°C, but no spore of 4–2 could grow at 43°C. Therefore, 46°C was chosen as the temperature for selection in the second round, which was 3°C higher than that of the first round. Then, more spores of 2–2 and 3–2 were grown at 46°C for 2 days till thermotolerant spores formed clones on the plates. A total of 580 clones generated by 2–2 and 680 clones generated by 3–2 were selected. Clones were grown at 30, 45, 46, 47, and 48°C. The number of highly thermotolerant spores generated by diploid 2–2, which were able to grow at 47°C or above, was much higher than by triploid 3–2 (Supplementary Figure S5). Twenty spores of 2-2 displaying the best thermotolerance at 48°C were named Dip2-1∼20 and those of 3–2 were named Tri2-1∼20. The thermal tolerance of these spores was investigated by a spot assay (Figure 1F). Dip2-1∼20 and Tri2-1∼20 exhibited the same growth as FIM1 at 30°C, while displaying better thermotolerance than FIM1 at higher temperatures. Results indicated that the frequency to obtain thermotolerant spores in the second round of screen was higher than that in the first round of screen. Meanwhile, the highest temperature allowed for growth in the second round of screen, as shown by Dip2-8, was 2°C higher than that in the first round of screen. The results suggested that an iterative screen based on the meiosis of diploid and triploid efficiently promoted the evolution of thermal tolerance.

The survival rates of spores growing at different temperatures were compared. In the first round of screen, the survival rates of spores produced by KM-Diploid, KM-Triploid and KM-Tetraploid at 45°C were 64.6, 57.5, and 27.6%, respectively, while those at 46°C were 21.8, 28.9, and 4.2%, respectively (Figure 1G). In the second round of screen, the survival rates of spores produced by diploid 2-2 and triploid 3-2 at 46°C were 94.3 and 83.3% respectively, those at 47°C were 72.4 and 52.7%, respectively, and those at 48°C were 22.6 and 5.9%, respectively (Figure 1H). In general, spores of diploid strain were more thermotolerant than those of triploid and tetraploid strains. The result suggested that the thermotolerance evolved faster in the meiosis of diploid strain than of triploid or tetraploid strain.

### The Thermal Tolerance of Spores Generated by a Two-Round Screen was Better Than by a One-Round Screen.

**FIGURE 2:**
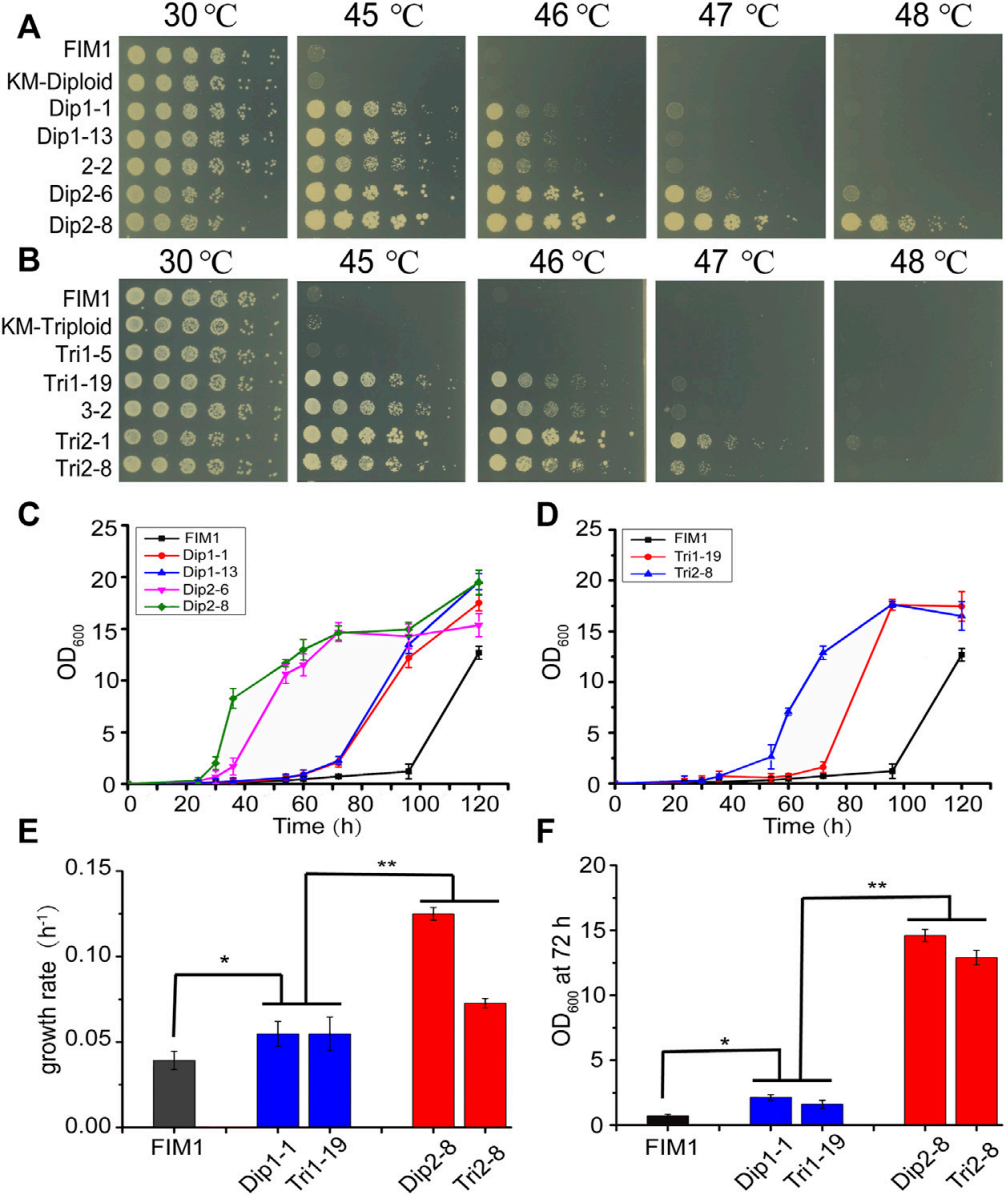
Comparison of the thermotolerant spores generated by diploid and triploid strains. **(A, B)** Spot assays of the thermotolerant spores generated by diploid strains **(A)** and triploid strains **(B)**. **(C, D)** The growth curves of the thermotolerant spores generated by diploid **(C)** and triploid strains **(D)** at 47°C. Values here and below represented mean ± SD (n = 3). **(E)** The maximum growth rate of representative spores generated by diploid and triploid strains at 47°C. **(F)** OD_600_ of representative spores at 72 h. The significant difference was measured by a Student’s *t*-test. *p < 0.05, **p < 0.01.

In the first round of screen, Dip1-1/Dip1-13 and Tri1-19 were the most thermotolerant spores generated by KM-Diploid and KM-Triploid, respectively. In the second round of screen, Dip2-8 and Tri2-1/Tri2-8 were the most thermotolerant spores produced by diploid 2–2 and triploid 3–2, respectively. The thermal tolerance of these spores was compared in spot assays (Figures 2A,B). Compared with the wild-type strain FIM1, the highest temperatures allowed for the growth of Dip1-13 and Tri1-19 were 46°C, that of Dip2-8 was 48°C, and those of Tri2-1 and Tri2-8 were 47°C. The results showed that Dip2-8 was the most thermotolerant spore, which was obtained in the two-round screen.

The growth curves of thermotolerant spores at 47°C were investigated. The lag phases of FIM1, Dip1-1, Dip1-13, Dip2-6 and Dip2-8 were 96, 60, 60, 34, 26 h, respectively (Figure 2C). The lag phases of Tri1-19 and Tri2-8 were 70 and 40 h, respectively (Figure 2D). The results suggested spores obtained in the second round of screen displayed shorter lag phases than in the first round screen. The maximum growth rates of spores from the second round of screen (Dip2-8 and Tri2-8) were significantly higher than those of spores from the first round of screen (Dip1-1 and Tri1-19), while maximum growth rates of first-round spores were significantly higher than that of FIM1 (Figure 2E). Similarly, values of OD_600_, which were indicators of the biomass, of the second-round spores at 72 h were significantly higher than those of the first-round spores, while values of OD_600_ of the first-round spores at 72 h were significantly higher than that of FIM1 (Figure 2F). The shorter the lag phase, the higher the growth rate and the higher biomass at the high temperature indicated the better thermal tolerance. Therefore, through the iterative cycle of “diploid/polyploid -meiosis -selection of spores at high temperature”, the thermal tolerance of spores generated by the two-round screen was significantly better than by the one-round screen, while the thermal tolerance of spores generated by the one-round screen was better than that of the original strain.

### *PSR1* and *PDE2* Negatively Regulated the Thermotolerance of *K. marxianus*

**FIGURE 3:**
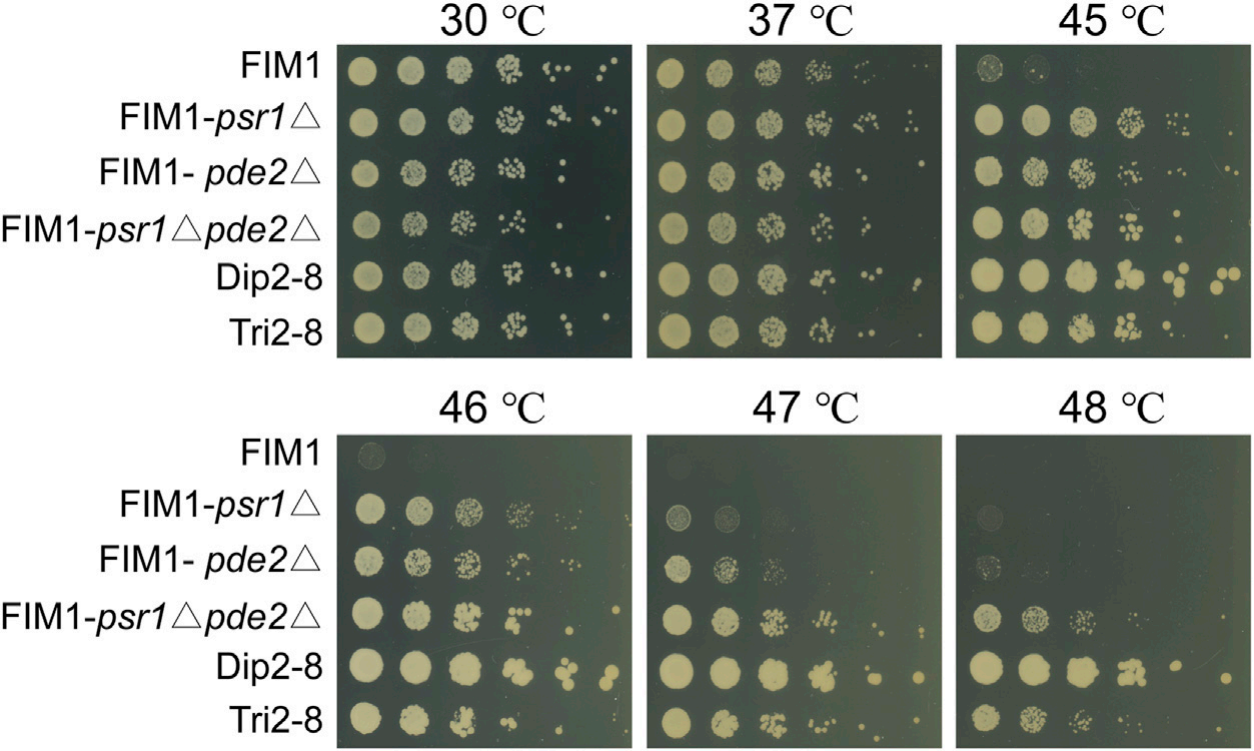
Spot assays of FIM1 carrying the deletion of *PSR1*, *PDE2* or a combination of both deletions. FIM1, Dip2-8 and Tri2-8 were spotted as controls.

To investigate the mutations underlying the thermal tolerance, Dip1-1, Dip1-13, Dip2-6, Dip2-8, Tri1-19 and Tri2-8 were subjected to the whole-genome sequencing. Seven mutations inside coding sequences, ten single-nucleotide polymorphisms (SNPs) and three insertion-deletions (InDels) inside non-coding sequences (NCDS) were identified in the spores (Table 2). Origins of some mutations could be traced back to ancestor strains (Table 2). For example, a frameshift in *PSR1* in Tri2-8 was inherited from Tri1-19. An insertion of “C” at 357288bp of chromosome 7 in all six spores was presumably inherited from KM-Diploid. Other SNPs and InDels might be caused by replication and repair errors during the meiosis or the vegetative growth afterwards. Notably, compared with the wild-type strain FIM1, Dip2-6 and Dip2-8 contained nonsense mutations of *PSR1* and *PDE2* genes, while Tri1-19 and Tri2-8 contained frameshift of *PSR1*. The result suggested mutations of *PSR1* and *PDE2* were the major contributors to the improved thermotolerance. To validate this idea, *PSR1* and *PDE2* were deleted individually or together in FIM1 to construct FIM1-*psr1*Δ, FIM1-*pde2*Δ and FIM1-*psr1*Δ*pde2*Δ strains, respectively (Supplementary Figure S6). These strains, along with Dip2-8, Tri2-8 and FIM1, were grown at 30°C, 37°C, 45°C, 46°C, 47°C, and 48°C. No significant growth difference was found between strains at 30°C and 37°C. The growth of FIM1-*psr1*Δ and FIM1-*pde2*Δ was significantly better than that of FIM1 at 45°C, 46°C and 47°C. The growth of FIM1-*psr1*Δ*pde2*Δ was better than that of FIM1-*psr1*Δ and FIM1-*pde2*Δ at 45, 46, and 47°C (Figure 3). These results suggested that *PSR1* and *PDE2* were key genes that negatively regulated the thermotolerance in *K. marxianus* and that two genes functioned in parallel pathways. However, the growth of FIM1-*psr1*Δ*pde2*Δ at 47°C and 48°C was poorer than that of Dip2-8, which contained five SNP and one InDel inside NCDS, besides the mutation of *PSR1* and *PDE2* (Table 2). The growth FIM1-*psr1*Δ at 47°C and 48°C was poorer than that of Tri2-8, which contained one InDel inside NCDS, besides the mutation of *PSR1* (Table 2). The result suggested that mutations inside NCDS, other than the mutations of *PSR1* and *PDE2*, also contributed to the thermotolerance.

**TABLE 2:** SNPs and InDels in thermotolerant spores.

Posi-tion^a^	Chromo-some	Mutation sites	ORFs	Change in nucleotide(s)	Change of amino acid	Strain	Origin of the mutation^b^
CDS	2	872630	*MUC1*	T1942G	Ser648 > Ala	Dip1-1	Dip1-1
	3	487709	*PSR1*	G256T	Glu86 > *Stop*	Dip2-6	Dip2-6
	3	1272805	*PDE2*	C1019A	Ser340 > *Stop*	Dip2-6	Dip2-6
	3	487709	*PSR1*	G256T	Glu86 > *Stop*	Dip2-8	Dip2-8
	3	1273144	*PDE2*	G680A	Trp227 > *Stop*	Dip2-8	Dip2-8
	3	487943	*PSR1*	+TAAAGAGG	*Frameshift*	Tri1-19	Tri1-19
	3	487943	*PSR1*	+TAAAGAGG	*Frameshift*	Tri2-8	***Tri1-19***
NCDS	7	357288	—	+C	—	Dip1-1	***KM-Diploid***
	3	1572771	—	+T	—	Dip1-13	Dip1-13
	7	357288	—	+C	—	Dip1-13	***KM-Diploid***
	1	1240060	—	C- > A	—	Dip2-6	Dip2-6
	8	620828	—	T- > G	—	Dip2-6	Dip2-6
	3	1572771	—	+T	—	Dip2-6	***Dip1-13***
	7	357288	—	+C	—	Dip2-6	***KM-Diploid***
	1	483871	—	G- > A	—	Dip2-8	Dip2-8
	1	541343	—	G- > A	—	Dip2-8	Dip2-8
	1	1182783	—	A- > T	—	Dip2-8	Dip2-8
	1	1240060	—	C- > A	—	Dip2-8	Dip2-8
	3	631197	—	G- > A	—	Dip2-8	Dip2-8
	7	357288	—	+C	—	Dip2-8	***KM-Diploid***
	1	204786	—	+A	—	Tri1-19	Tri1-19
	3	1572771	—	+T	—	Tri1-19	Tri1-19
	7	357288	—	+C	—	Tri1-19	***KM-Diploid***
	7	357288	—	+C	—	Tri2-8	***KM-Diploid***

a CDS, was short for coding sequence and NCDS, was for non-coding sequence.

b Name of the ancestor strain from which the mutation was inherited was in bold.

## Discussion

High yielding of heterologous proteins, including β-glucosidase (Su et al., 2021), feruloyl esterase (Liu et al., 2018), and virus-like particles (Duan et al., 2019), has been successfully achieved in *K. marxianus*. Meanwhile, superior features of fast growth, thermotolerance and the capacity to assimilate pentose facilitate the production of bioethanol and chemicals in *K. marxianus* (Suzuki et al., 2019). Collectively, *K. marxianus* is a microbial cell factory with great potentials.

Improving the thermal tolerance of *K. marxianus* is a necessity to promote its industrial applications, especially in the production of ethanol. Based on the genetic diversity produced by the recombination of meiosis, we established an iterative cycle of “diploid/polyploid - meiosis - selection of spores at high temperature” to improve the thermal tolerance of *K. marxianus*. During the vegetative growth of yeast cells, the genome-wide single-nucleotide mutation rate was at 2–3 × 10^−10^ per base per generation (Lynch et al., 2008; Zhu et al., 2014), and continuous culturing yeast cells at high temperature were expected to enrich mutations that improved thermal tolerance of cells. Consistent with this idea, the growth rate of *S. cerevisiae* at 40°C was increased by 1.57 times, through the continuous passage of the culture at 39.5°C for more than 90 days (Caspeta et al., 2014). During meiosis, DNA repair associated recombination was mutagenic, providing extra genetic diversity that can contribute to adaptive evolution (Rattray et al., 2015). In this study, we established an iterative cycle of “diploid/polyploid - meiosis - selection of spores at high temperature” in *K. marxianus*, and successfully improved the maximum temperature allowed for growth by 3°C after only two rounds of the screen. Furthermore, the average concentration of ethanol produced by second-round spores (Dip 2-8 and Tri2-8) at 45°C was significantly more than that by one-round spores (Dip1-1 and Tri1-19) (Supplementary Figure S7). The result suggested the ethanol productivity at high temperatures was also improved during the iterative cycle of evolution.

Compared with the evolution of vegetatively growing cells, one obstacle to the meiosis-based evolution was the requirement of constructing diploid/polyploid strains for each round of screen. In some cases, spores displaying better thermotolerance could not be selected to construct diploid/polyploid strains because mating types of spores were not compatible. For example, Tri1-4 (*MAT*α/α) was more thermotolerant than Tri1-5 (*MAT*a/a), but the latter was selected to construct a triploid strain as it was compatible with Tri1-19 (*MAT*α) (Figure 1E, Supplementary Figure S3). Therefore, to construct diploid/triploid from the most thermotolerant spores, a convenient protocol to switch the mating type needed to be established in the following study. In some cases, the mating type was not consistent with the ploidy. For example, the mating type of Dip1-2 was identified as *MAT*a/α by PCR (Supplementary Figure S3). However, Dip1-2 was identified to be a haploid strain in the flow cytometry analysis. Meiosis might result in rearrangement at the *MAT* locus and lead to the production of multiple *MAT* loci in the genome.

In this study, diploid, triploid and tetraploid strains were selected for the iterative screen. The proportion of thermotolerant spores from the diploid was higher than that from the triploid, and that from the triploid was higher than that from the tetraploid (Figures 1E,F). This result might be related to the fact that the thermal tolerance of *K. marxianus* decreased with increased ploidies (Figure 1D). The same results were reported for *S. cerevisiae* (Zhang et al., 2017). In addition, aneuploidy spores generated by the triploid strain might lead to an imbalance of cellular energy metabolism, which was detrimental to stress resistance (Torres et al., 2007). The growth of aneuploid cells at high temperatures imposed an extra burden on the chromosome segregation and might cause a higher frequency of chromosome rearrangements and loss, which reduced the viability of progenies and efficiency of adaptive evolution (Yona et al., 2012).

Thermotolerant spores obtained in this screen, including Dip1-1, Dip1-13, Dip2-6, Dip2-8, Tri1-19 and Tri2-8, grew as well as wild-type FIM1 at 30°C. Whole-genome sequencing of these spores obtained in our screen did not reveal any rearrangement or loss of chromosome fragments, suggesting the natural chromosome structures of *K. marxianus* were required for the balance of normal growth at the regular temperature and improved growth at high temperatures. Four out of six thermotolerant spores contained mutations of *PSR1*, while two spores contained mutations of *PDE2*. FIM1 cells carrying an individual deletion of *PSR1* or *PDE2* exhibited increased thermotolerance, while deletion of both genes displayed additive effects on the thermotolerance. The result indicated that *PSR1* and *PDE2* negatively regulated the growth of *K. marxianus* at high temperatures (up to 48°C) through parallel pathways. In *S. cerevisiae*, *PDE2* encodes a high-affinity cAMP phosphodiesterase that catalyzes the degradation of cAMP, and thus negatively regulates numerous cAMP-dependent pathways (Sass et al., 1986). Psr1 is a membrane-associated phosphatase. Psr1 and its partner Psr2 form a complex with Whi2 to negative regulate TORC1, which is a signalling complex regulating the response to nutrients (Kaida et al., 2002; Chen et al., 2018). Notably, deletion of *PDE2* and *PSR1* reduced the viability of the cells after a transient heat shock, probably through constitutively activated cAMP-dependent pathways and TORC1 pathways, respectively (Jones et al., 2004; Teng et al., 2011). The results indicated *PSR1* and *PDE2* were positive regulators of the response to heat shock in *S. cerevisiae*, which looks like contradicting the results in *K. marxianus*. However, the mechanism supporting the survival of short exposure to extremely high temperatures differs that regulating growth at high temperatures. For example, Hsp104 plays a vital role in helping cells survive short-term heat shock, but is not required for the growth at high temperatures (Lindquist and Kim, 1996). Besides, there was no direct proof indicating that *PSR1* and *PDE2* were required for continuous growth at high temperatures in *S. cerevisiae*. *K. marxianus* is more thermotolerant than *S. cerevisiae* (Fonseca et al., 2008). It is possible that complex networks regulated by *PSR1* and *PDE2* in *K. marxianus*, such as PKA and TORC1 pathways, are rewired during the evolution to negatively regulate the growth at high temperatures. The detailed mechanism is worthwhile to be investigated in the following study.

## Data Availability

The original contributions presented in the study are publicly available. This data can be found here: https://www.ncbi.nlm.nih.gov/bioproject/PRJNA732519.
