# Supplementary material for: Meiosis-Based Laboratory Evolution of the Thermal Tolerance in Kluyveromyces marxianus
Source: Front Bioeng Biotechnol. 2022 Jan 11;9:799756. doi: 10.3389/fbioe.2021.799756 (PMC8786734; doi:10.3389/fbioe.2021.799756)
Supplement: Supplementary file 1 [file DataSheet1.docx]

**Table S1 Primers used in this study**

| Targets | Applications | Names | Sequences (5’-3’) |
| --- | --- | --- | --- |
| *PSR1* | Deletion by CRISPR/Cas9 | 1921U-F | TGGCTTGAATGAAGGACAGGTAG |
|  |  | 1921U-R | CAACGTTAAATGGTCAAAAGATCCATTAAACTCTCTAAAATCCTCCTATCTAATCC |
|  |  | 1921D-F | GGATTAGATAGGAGGATTTTAGAGAGTTTAATGGATCTTTTGACCATTTAACGTTG |
|  |  | 1921D-R | TATGGAGAGCTAAGGCATGGG |
|  |  | 1921Pam-F | TCATTTATCACCTCTTTATTGTG |
|  |  | 1921Pam-R | AACCACAATAAAGAGGTGATAAA |
|  |  | 1921JD-F | CCGGGAGCAGATATTCCATCA |
|  |  | 1921JD-R | CAAGTCAAACACGGTCACTCA |
| *PDE2* | Deletion by CRISPR/Cas9 | 2290U-F | AGCTCCCTCTGCCTGTCACC |
|  |  | 2290U-R | GTTAATCGATTTACCGTGATAACCTTTTTTCGAGCACTTCTTTGTTATACCG |
|  |  | 2290D-F | CGGTATAACAAAGAAGTGCTCGAAAAAAGGTTATCACGGTAAATCGATTAAC |
|  |  | 2290D-R | TTAAAACTGCTCTCGGTCCGA |
|  |  | 2290Pam-F | TCAGCAGTCCATGTATGTTGCAT |
|  |  | 2290Pam-R | AACATGCAACATACATGGACTGC |
|  |  | 2290JD-R | AAAGCCAATCTGCAACTTCAG |
| *HMR*a | Amplification of the ORF | W21F | ATCAGAATCACGCAAAGGGACGT |
|  |  | W21R | AACACGTCCCTTTGCGTGATTCT |
|  |  | W22F | GATCGCCTTCAGAAGCTTAGTTCT |
|  |  | W22R | AAACAGAACTAAGCTTCTGAAGGC |
| *HMR*a | Target deletion by CRISPR/Cas9 | W23F | GCCTAGAATTGATGGGGCATA |
|  |  | W23R | TTCTTGGGCTATTTGATTTTTTCAC |
|  |  | W24F | ACTAGTGCTTAGTTCTCTTGTAGTACTGCGT |
|  |  | W24R | GCAGAGACTGGCACAAATCTCGGTT |
|  |  | W29F | GATCACGTGAATAGTCTATTGGGAA |
|  |  | W29R | GTTACGCAAAACTTCCTCCCTTGGA |
| *HML*α | Amplification of the ORF | W25F | ATCTCGATCATGCAGATCATATG |
|  |  | W25R | AACCATATGATCTGCATGATCGA |
|  |  | W26F | GATCATAGAACGTCGTTCGGTTGA |
|  |  | W26R | AAACTCAACCGAACGACGTTCTAT |
| *HML*α | Deletion by CRISPR/Cas9 | W27F | GTTTCTACTGGTGACCGTGTTCAGA |
|  |  | W27R | GCATGATCGAAATTACTTTTCAAGT |
|  |  | W28F | ACTAGTTGAGTTTGCGGAAGAGGAATATGAG |
|  |  | W28R | GTGGCCTATATAATGGCATAATCAA |
|  |  | W30F | GAAGAAAGAGGATGGATCATACGCG |
|  |  | W30R | GATGCGGTGGCTCCTCGACAAACCT |
| *MAT*a | Deletion by CRISPR/Cas9 | W15F | GATCTCTTAAACACTTTACCAGAT |
|  |  | W15R | AAACATCTGGTAAAGTGTTTAAGA |
|  |  | W20F | ATCTATTTAATAGGACCAGCTAT |
|  |  | W20R | AACATAGCTGGTCCTATTAAATA |
| *MAT*α | Deletion by CRISPR/Cas9 | W35F | GATCATGCATTCTAATAATAAAAA |
|  |  | W35R | AAACTTTTTATTATTAGAATGCAT |
|  |  | W36F | ATCTATTTTTGATTAAAAATAAA |
|  |  | W36R | AACTTTATTTTTAATCAAAAATA |
| *MAT*a | Amplification of the *MAT*a locus | YY270F | TGCAACCAACCAATCCCTTCCAAATTC |
|  |  | YY271F | TCTTCCTTGAACCCGAAGCAAAAGATC |
| *MAT*α | Amplification of the *MAT*α locus | YY270F | TGCAACCAACCAATCCCTTCCAAATTC |
|  |  | YY272F | AACTTCAATCCCCGACCCACCGCAGTC |

**Table S2 Plasmids used in this study**

| **Names** | **Applications** | **Sources** |
| --- | --- | --- |
| ARS1-CRISPR | Vector for co-expression of gRNA and Cas9, containing ARS1 and *URA3* | 1 |
| LHZ328 | Co-expression of gRNA and Cas9 to delete *HMR*a locus | This study |
| LHZ329 | To provide the donor sequence for the deletion of *HMR*a locus | This study |
| LHZ330 | Co-expression of gRNA and Cas9 to delete *HML*α locus | This study |
| LHZ331 | To provide the donor sequence for the deletion of *HML*α locus | This study |
| LHZ326 | Co-expression of gRNA and Cas9 to transform *MAT*a into *MAT*α | This study |
| LHZ327 | Co-expression of gRNA and Cas9 to transform *MAT*α into *MAT*a | This study |
| CRISPR-1921 | Co-expression of gRNA and Cas9 to delete *PSR1* | This study |
| CRISPR-2290 | Co-expression of gRNA and Cas9 to delete *PDE2* | This study |
| pUKDN127-Kan | pKD1, *KanMX6* | This study |
| pUKDN127-Hyg | pKD1, *hphMX4* | This study |

1. Liu et al. *Microb Cell Fact* (2018) 17:144


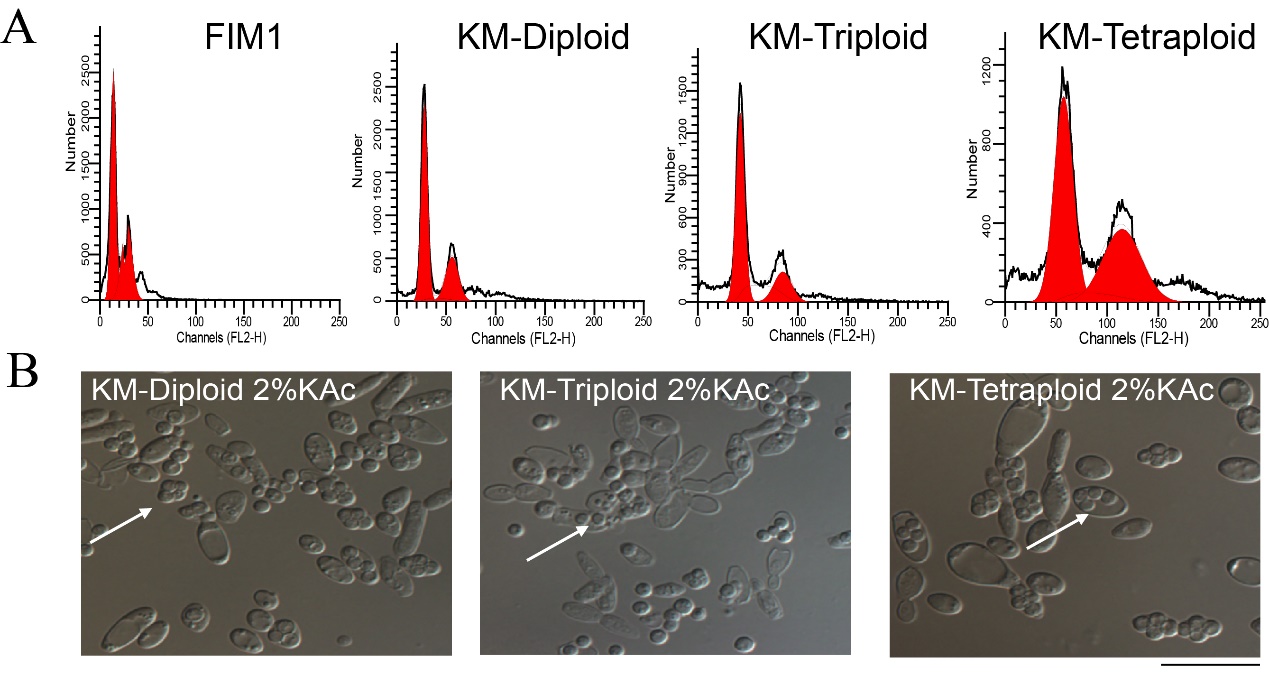


**Figure S1** DNA contents and meiosis of diploid and polyploid strains for the first round of screen. (A) Analysis of DNA contents of FIM1, KM-Diploid, KM-Triploid and KM-Tetraploid by flow cytometry. (B) Spores formed during the meiosis. Spores were indicated by white arrows. Scale bar: 10 μm.


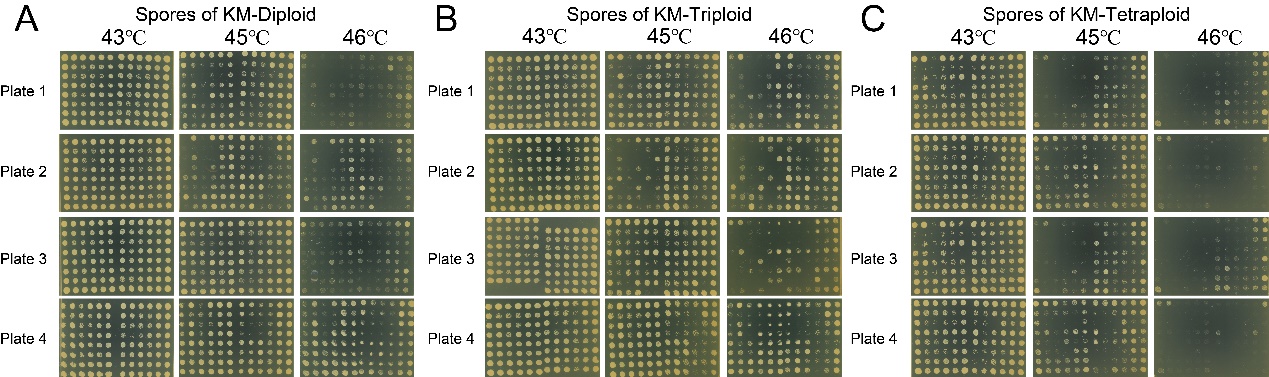


**Figure S2** The thermal tolerance of spores generated in the first round of screen. ((A-C) The thermal tolerance of ~392 spores generated from KM-Diploid (A), KM-Triploid (B) and KM-Tetraploid strain (C). Spores were grown in YPD plates at indicated temperatures for 2 days.


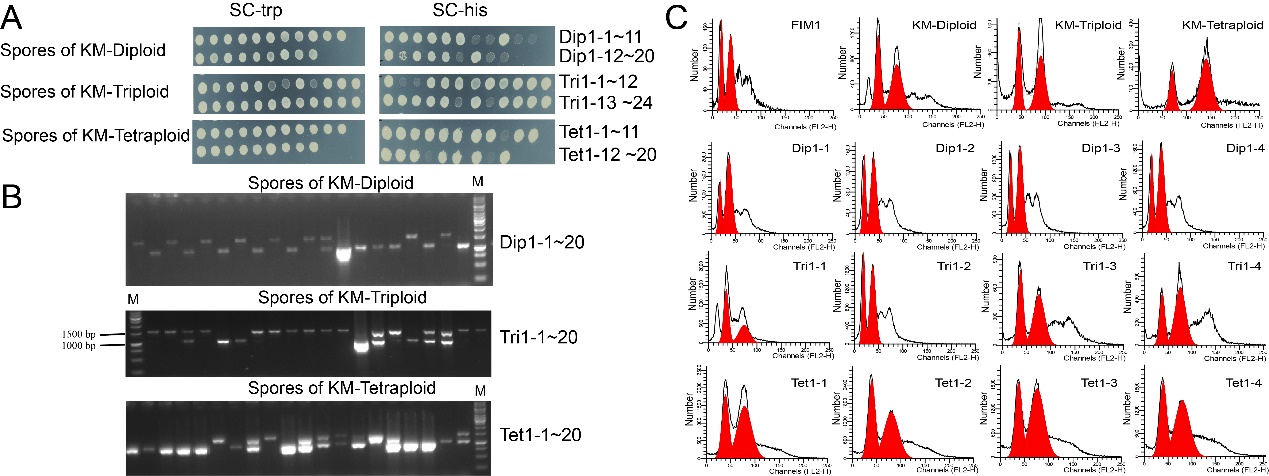


**Figure S3** Analysis of auxotrophic markers, mating types and ploidies of spores generated in the first round of screen. (A) Analysis of auxotrophic markers. Spores were replicated by a pinpad onto SC-trp and SC-his plates. (B) Analysis of mating types by PCR. *MAT*a locus produced a band of 1062 bp and *MAT*α locus produced a band of 1515 bp. M:1 kb DNA Ladder. (C) Analysis of DNA contents of spores by flow cytometry.


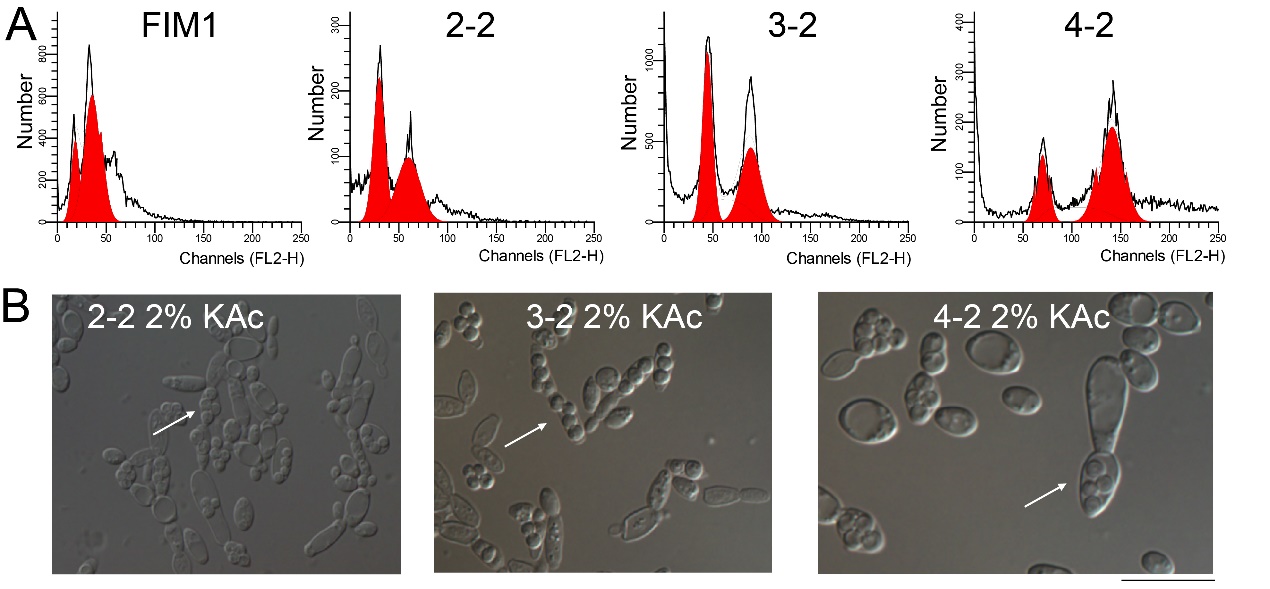


**Figure S4** DNA contents and meiosis of diploid and polyploid strains for the second round of screen. (A) Analysis of DNA contents of FIM1, 2-2, 3-2, 4-2 by flow cytometry. (B) Spores formed during the meiosis. Spores were indicated by white arrows. Scale bar: 10 μm


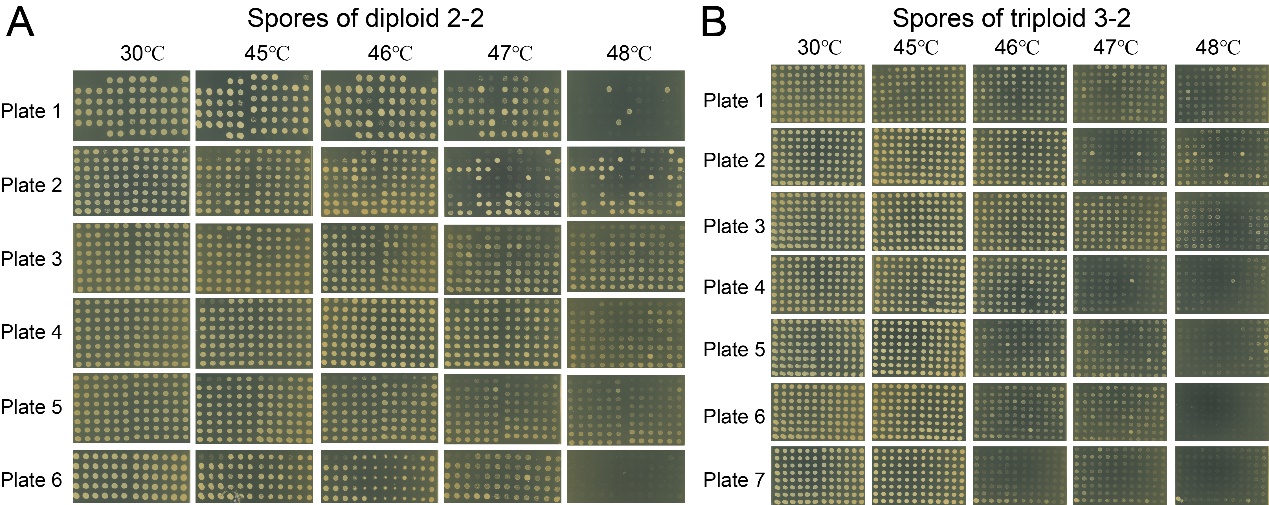


**Figure S5** The thermal tolerance of spores generated from diploid 2-2 and triploid 3-2 in the second round of screen. (A, B) The thermal tolerance of spores generated from 2-2 (A) and 3-2 (B). Spores were grown in YPD plates at indicated temperatures for 2 days.


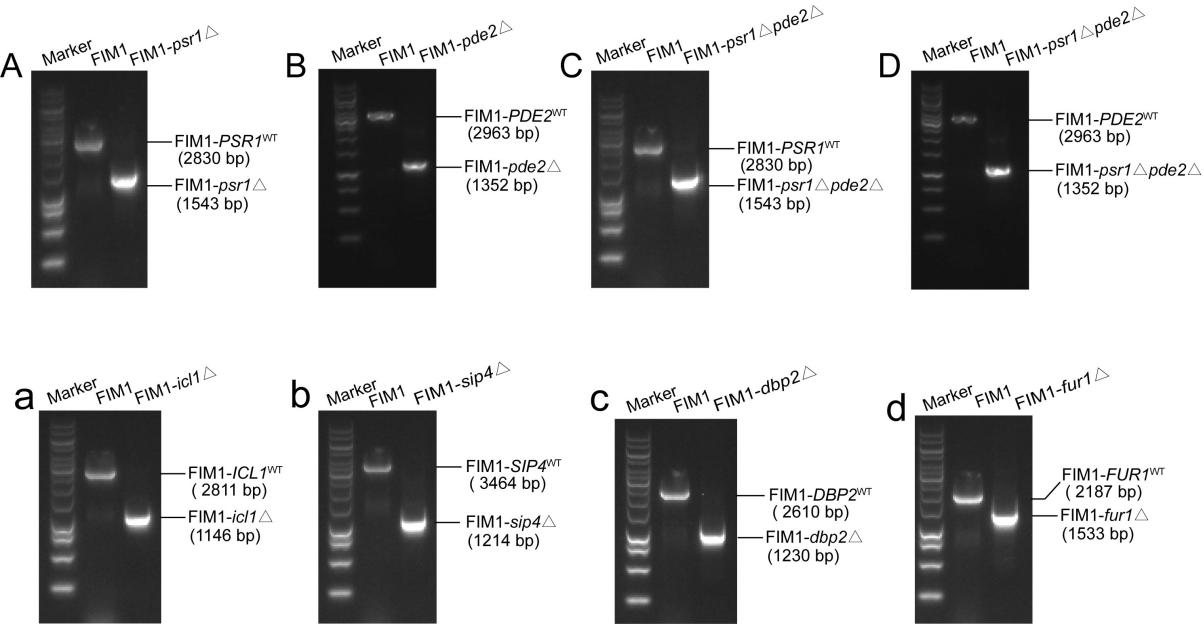


**Figure S6** Identification of deletions of *PSR2* and *PDE2*. (A, C) Identification of the deletion of *PSR1* in FIM1-*psr1*Δ (A) and FIM1-*psr1*Δ*pde2*Δ (C) strains by PCR . (B, D) Identification of the deletion of *PDE2* in FIM1-*pde2*Δ (B) and FIM1-*psr1*Δ*pde2*Δ (D) strains by PCR. Marker：1 kb DNA Ladder.


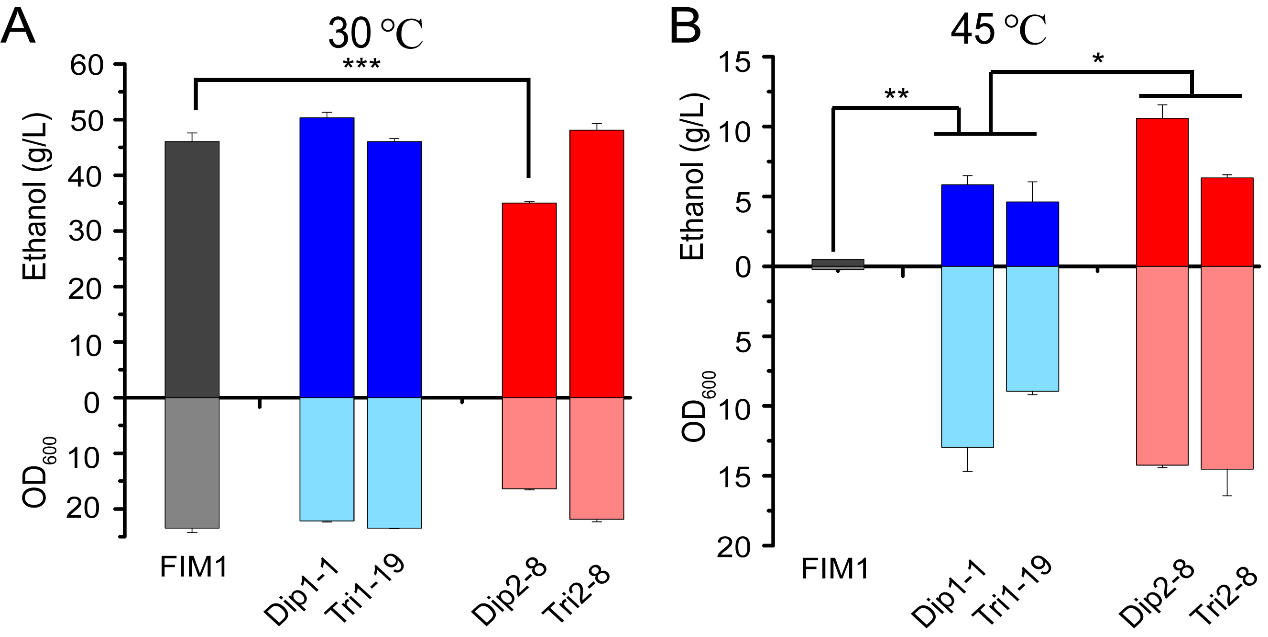


**Figure S7** Ethanol productivities of thermotolerant spores. Fresh colonies of FIM1, Dip1-1, Dip2-8, Tri1-19 or Tri2-8 were inoculated separately into 50 mL high-glucose YPD liquid medium (10 g/L yeast extract, 20 g/L hipolypepton, 200 g/L glucose) and were grown at 30 ℃ (A) or 45 ℃ (B) for 72 h. Concentrations of ethanol in cultures were measured by HPLC as described before (Lan et al. *Biotechnology for Biofuels* (2021) 14:220). OD_600_ of cultures at 72 h were shown below. Values represented mean ± SD (n=3). The significant difference was measured by a Student’s *t*-test. *p < 0.05, **p < 0.01
